# Value of arterial blood gas analysis in patients with acute dyspnea: an observational study

**DOI:** 10.1186/cc10268

**Published:** 2011-06-09

**Authors:** Emanuel Burri, Mihael Potocki, Beatrice Drexler, Philipp Schuetz, Alexandre Mebazaa, Ulrike Ahlfeld, Catharina Balmelli, Corinna Heinisch, Markus Noveanu, Tobias Breidthardt, Nora Schaub, Tobias Reichlin, Christian Mueller

**Affiliations:** 1 Department of Internal Medicine, University Hospital Basel, Petersgraben 4, Basel, 4031, Switzerland; 2 Department of Gastroenterology, University Hospital Basel, Petersgraben 4, Basel, 4031, Switzerland; 3 Department of Anesthesiology and Critical Care Medicine, Université Paris Diderot and Hospital Lariboisière, 2, rue Ambroise - Paré, 75475 Paris Cedex 10, France

## Abstract

**Introduction:**

The diagnostic and prognostic value of arterial blood gas analysis (ABGA) parameters in unselected patients presenting with acute dyspnea to the Emergency Department (ED) is largely unknown.

**Methods:**

We performed a post-hoc analysis of two different prospective studies to investigate the diagnostic and prognostic value of ABGA parameters in patients presenting to the ED with acute dyspnea.

**Results:**

We enrolled 530 patients (median age 74 years). ABGA parameters were neither useful to distinguish between patients with pulmonary disorders and other causes of dyspnea nor to identify specific disorders responsible for dyspnea. Only in patients with hyperventilation from anxiety disorder, the diagnostic accuracy of pH and hypoxemia rendered valuable with an area under the receiver operating characteristics curve (AUC) of 0.86. Patients in the lowest pH tertile more often required admission to intensive care unit (28% vs 12% in the first tertile, P < 0.001) and had higher in-hospital (14% vs 5%, P = 0.003) and 30-day mortality (17% vs 7%, P = 0.002). Cumulative mortality rate was higher in the first (37%), than in the second (28%), and the third tertile (23%, P = 0.005) during 12 months follow-up. pH at presentation was an independent predictor of 12-month mortality in multivariable Cox proportional hazard analysis both for patients with pulmonary (P = 0.043) and non-pulmonary disorders (P = 0.038).

**Conclusions:**

ABGA parameters provide limited diagnostic value in patients with acute dyspnea, but pH is an independent predictor of 12 months mortality.

## Introduction

Patients presenting to the emergency department (ED) with acute dyspnea require a rapid diagnostic work up to decide whether hospitalization or intensive care admission are needed and to guide further therapy [1]. Acute heart failure (AHF), exacerbation of chronic obstructive pulmonary disease (COPD), and pneumonia account for the majority of emergency consultations by patients with acute dyspnea [2,3]. As dyspnea is not a specific symptom, the rapid and accurate identification of the underlying causes remains a clinical challenge. Misdiagnosis causes morbidity and increases time to discharge and treatment cost [4]. In addition, treatment for one common disorder, e.g. AHF, might even be hazardous for patients with other conditions such as exacerbated COPD or pneumonia [5].

At presentation to the ED, arterial blood gas analysis (ABGA) is often performed in dyspneic patients to assess acid-base disturbances, and to diagnose and quantify respiratory insufficiency. Accordingly, it has been recommended for the clinical work-up in several dyspnea-related diseases [6-9]. Several studies have investigated the value of ABGA in patients with suspected pulmonary embolism (PE) [10-12], but the usefulness of the different prediction rules proposed by theses studies has been questioned [13]. In patients with community-acquired pneumonia (CAP), Levin et al. examined factors associated with the use of ABGA and also assessed whether measurement of ABGA in patients was associated with hospitalization, ICU treatment, or death [14].

The role of ABGA in unselected patients with acute dyspnea, however, is poorly studied. Specifically, it is unknown whether ABGA parameters can be used as a diagnostic marker in patients with a non-specific symptom such as acute dyspnea. Additionally, it should be further investigated whether the prognostic value of ABGA parameters observed in patients with exacerbated COPD and pneumonia can be expanded to unselected patients with acute dyspnea.

The aim of this study was to prospectively investigate the value of ABGA parameters as biological markers for diagnosis and prognosis in patients presenting to the ED with acute dyspnea.

## Materials and methods

### Setting and study population

In this prospective observational study, we investigated patients presenting to the ED of the University Hospital Basel, Switzerland, with acute dyspnea. If several symptoms were present, dyspnea had to be the primary complaint. The interdisciplinary ED manages around 40,000 patients per year. It is an independent department with its own senior staff and rotating physicians from both the internal medicine department and surgery department. A total of 1,135 patients were enrolled in two series of consecutive patients: 452 patients (out of 665 patients screened) were enrolled from May 2001 to April 2002 in the B-type natriuretic peptide for Acute Shortness of Breath Evaluation (BASEL) study [2], and another 683 patients (of 765 patients screened) were enrolled between April 2006 and March 2008. Patient recruitment had to be paused between 2003 and 2005 due to a lack of resources. Exclusion criteria were identical during both recruitment periods: age younger than 18 years, an obvious traumatic cause of dyspnea, cardiogenic shock, severe renal disease (defined as serum creatinine level of more than 250 μmol/l in the first series and by hemodialysis in the second period). Patients were enrolled by a study physician who was otherwise not involved in the clinical management of the individual patient. Of the 1,153 patients enrolled, 530 had ABGA at presentation and were considered as the study population. The study was carried out according to the principles of the Declaration of Helsinki and approved by the local ethical committee. Written informed consent was obtained from all participants prior to study entry.

### Adjudication of the final diagnosis

After patient discharge from the hospital, the final diagnosis was independently adjudicated in a blinded fashion by two internal medicine specialists who were not involved in the care of the patients during the hospitalization according to current recommendations on the basis of available medical records, including B-type natriuretic peptide (BNP) levels, the results of all diagnostic investigations, the response to treatment, and autopsy data in those patients who died [1,8,15]. The physicians adjudicated the final diagnosis by choosing one or more diagnoses from a pre-specified list that included the following items: AHF, exacerbated COPD/asthma, CAP/bronchitis, PE, hyperventilation from anxiety disorder (HV), other, or unknown. If more than one cause for acute dyspnea was identified, the leading disorder responsible for the current episode of acute dyspnea was determined. When there was disagreement about the final diagnosis, cases were reviewed and adjudicated in conjunction with a third internal medicine specialist who was considered an expert in the field.

### Blood sampling and laboratory methods

All patients underwent an initial clinical assessment that included history taking, a physical examination, non-invasive blood pressure measurement, 12-lead ECG, continuous ECG-monitoring, pulse oximetry, standard blood tests, and chest radiography. ABGA samples were taken immediately after presentation to the ED after the initial assessment from the attending physician. If multiple samples were taken, only values from the first were included in the analysis. The decision to perform ABGA was made solely by the physician in charge, based on clinical grounds. If oxygen supply had been started prior to hospital entry, it was stopped for at least two minutes before collection of the sample. For ABGA, a 1 ml arterial specimen of blood was collected from a radial artery into heparinized syringes and immediately analyzed using the Radiometer ABL™ 700 (Radiometer Medical ApS, Copenhagen, Denmark). The analysis included measurement of pH, the partial pressure of arterial carbon dioxide (PaCO_2_), and the partial pressure of arterial oxygen (PaO_2_) based on ion-selective electrodes with potentiometric measurement for pH and PaCO_2_, and amperometric measurement of PaO_2_. Standard bicarbonate (HCO_3_) was calculated from the observation parameters pH and PaCO_2_. The Radiometer ABL™ 700 has a high accuracy and repeatability (precision within run: pH 0.0020, for PaCO_2 _0.40 mmHg, and PaO_2 _0.9 mmHg; precision between run: pH 0.0084, PaCO_2 _0.66 mmHg, and PaO_2 _1.5 mmHg). Values of PaO_2 _above 10.8 kPa, PaCO_2 _4.7 to 6.1 kPa, pH 7.35 to 7.45, and HCO_3 _21 to 26 mmol/l were considered within normal ranges. To calculate the estimated glomerular filtration rate the abbreviated 4-variable Modification of Diet in Renal Disease study equation was used.

### Endpoints and follow up

The diagnostic value of ABGA variables was assessed in comparison to the adjudicated gold standard diagnosis. The prospective value of ABGA variables was assessed for the prediction of short-term events, ICU admission, in-hospital mortality, and 30-day mortality, as well as for long-term outcome. All patients were contacted by telephone interview performed by trained researchers blinded to the results of laboratory testing. In case of uncertainties regarding vital status, referring physicians and administrative database of the respective hometown were contacted.

### Statistical analysis

Categorical variables are presented as numbers and percentage, continuous variables as mean ± standard deviation (SD) or median and 95% confidence interval (95% CI) or interquartile range (IQR). Comparisons were made using the Student's t-test, Mann-Whitney U test, Wilcoxon test, and Kruskal-Wallis test for numerical parameters and the chi-square test for categorical data where appropriate. Stepwise multiple logistic regression analysis was used to identify ABGA parameters that were independently correlated to a defined endpoint. HCO_3 _values were calculated from pH and PaCO_2 _and therefore not included in the regression model. (ROC) operating characteristics curves were calculated to determine the sensitivities, specificities, likelihood ratios, and predictive values for all independent ABGA parameters. Kaplan-Meier analysis was performed for survival and log-rank values to assess statistical significance. We used Cox proportional hazard models adjusted for age, sex, New York Heart Association (NYHA) class, history of coronary artery disease, COPD, any pulmonary disease or chronic kidney disease, smoking status, previous use of oral diuretics or inhaled beta agonists, systolic blood pressure, respiration rate, body mass index (BMI), more than one cause of dyspnea and pH at admission to compute hazard ratios (HR) and 95% CI of predictors of 12-month mortality. All hypothesis testing was two-tailed, and a *P*-value of 0.05 was considered statistically significant. Analyses were performed using SPSS (Release 19.0.0, SPSS Inc., Chicago, IL, USA) and MedCalc for Windows (Version 11.4.4, Mariakerke, Belgium).

## Results

### Characteristics of study population

Among the study population (*n *= 530), AHF was the adjudicated diagnosis in 206 patients (39%). Among the non-cardiac causes of dyspnea, exacerbated COPD/asthma was present in 118 patients (22%), CAP/bronchitis in 94 patients (18%), PE in 28 patients (5.3%), HV in 18 patients (3.4%), and other causes such as pneumothorax, malignancy, interstitial lung disease, or anemia in 66 patients (13%). In 11 patients (2.1%), the diagnostic work up was insufficient to adjudicate a diagnosis. One hundred and fourteen patients (22%) presented with more than one cause responsible for acute dyspnea. Among those, 56 patients (49%) had multiple pulmonary disorders. Patients in the study population more often were male, were obese, presented with higher NYHA class, were current or former smokers, had a history of a pulmonary disease and accordingly more frequently used inhaled beta agonists and inhaled and oral steroids, more often had clinical signs of respiratory tract infection, had lower oxygen saturation rates, and lower BNP values. In the study population, patients more often suffered from exacerbated COPD, asthma, bronchitis, or pneumonia and more often were admitted to the hospital and to ICU. The detailed baseline characteristics are summarized in the supplemental digital content in Additional file 1.

### Diagnostic accuracy of arterial blood gas parameters

**Figure 1:**
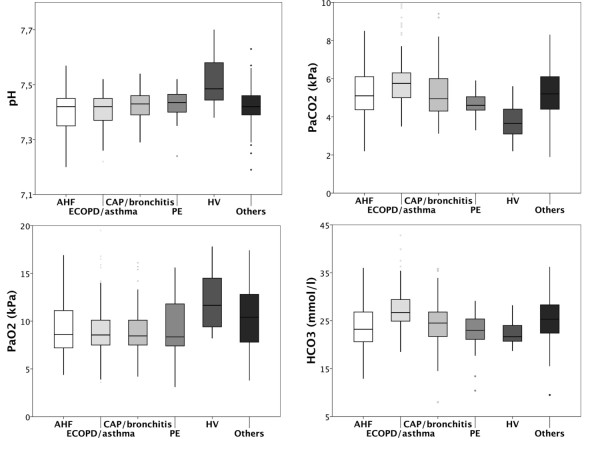
**Values of arterial blood gas parameters at presentation**. Boxplots (median, interquartile range) of pH (upper left), partial pressure of arterial carbon dioxide (PaCO_2_; upper right), partial pressure of arterial oxygen (PaO_2_; lower left), and partial pressure of bicarbonate (HCO_3_; lower right) at presentation in patients with acute heart failue (AHF), excerbated chronic obstructive pulmonary disease or asthma (ECOPD/asthma), community-acquired pneumonia (CAP), pulmonary embolism (PE), hyperventilation from anxiety (HV), and other disorders.

Values of ABGA parameters at presentation varied widely in all underlying disorders (Figure 1). Acidosis (pH < 7.35) was found in 88 patients (17%) at presentation. Of those, 9 (10%) had metabolic acidosis, 66 (75%) respiratory acidosis, and 13 (15%) mixed-type acidosis. In 142 patients (27%) alkalosis (pH > 7.45) was present at presentation. Six patients (3.4%) showed metabolic, 108 (60%) respiratory, and 65 (36%) mixed-type alkalosis. Hypoxemia (PaO_2 _< 10.8 kPa) was found in 377 patients (71%).

Evaluating the value of AGBA parameters at presentation as a diagnostic test, we found that neither the presence of respiratory or metabolic acidosis or alkalosis nor individual or combined absolute values of ABGA parameters were able to reliably identify specific disorders responsible for dyspnea or to distinguish patients with pulmonary disorders from other causes of dyspnea (Table 1). Only in patients with hyperventilation from anxiety disorders, pH (odds ratio (OR) 4.50, 95% CI 2.27 to 8.92) and hypoxemia (OR 0.21, 95% CI 0.07 to 0.65) provided useful diagnostic information. We found a combined area under the curve of 0.86 (95% CI 0.82 to 0.88) with an optimal cut-off for pH of 7.45. Using this cut-off, pH yielded a sensitivity and specificity of 72% and 75%, respectively. When subgroups of patients with NYHA III/IV (*n *= 471, 89%), or with oxygen saturation below 90% (*n *= 117, 23%) were analyzed separately, the diagnostic accuracy did not increase.

**Table 1:** Diagnostic value of ABGA parameters to identify disorders responsible for acute dyspnea (only significant variables)

	Variable	OR (95% CI)	AUC (95% CI)
**Acute heart failure**	pH	0.58 (0.44-0.77)	0.615 (0.57-0.66)
	PaCO_2_	0.81 (0.69-0.95)	
**Exacerbated COPD or asthma**	pH	1.64 (1.14-2.37)	0.670 (0.63-0.71)
	PaCO_2_	1.44 (1.20-1.73)	
	Hypoxemia	1.71 (1.04-2.82)	
**Pulmonary embolism**	PaCO_2_	0.61 (0.44-0.85)	0.678 (0.64-0.72)
**Pneumonia or bronchitis**	Hypoxemia	1.84 (1.07-3.17)	0.558 (0.51-0.60)
**Anxiety disorder**	pH	4.50 (2.27-8.92)	0.855 (0.82-0.88)
	Hypoxemia	0.21 (0.07-0.65)	
**Pulmonary disorder**	pH	1.40 (1.06-1.87)	0.639 (0.60-0.66)
	PaCO_2_	1.31 (1.12-1.54)	
	Hypoxemia	2.01 (1.37-3.13)	

Multivariable logistic regression analysis. Odds ratios (OR) with 95% confidence interval (95% CI) for independent arterial blood gas analysis (ABGA) parameters; Categorial variables included in the model were hypoxemia, metabolic acidosis or alkalosis, respiratory acidosis or alkalosis, mixed-type alkalosis or acidosis, pH, partial pressure of arterial carbon dioxide (PaCO_2_), and partial pressure of arterial oxygen (PaO_2_). OR are given for any increase of 1 kPa for PaO_2 _or PaCO_2_, and 0.1 for pH. Area under the curve (AUC) quantifying the diagnostic accuracy of the individual variables in settings with only one significant diagnostic ABGA variable (pulmonary embolism and community-acquired pneumonia), and for the optimal combination of ABGA variables in settings with more than one significant variables (all others). COPD, chronic obstructive pulmonary disease.

### Value of ABGA parameters to predict admission to ICU

Of the study population (*n *= 530), 91 patients (17%, AHF = 46, exacerbated COPD/asthma = 14, CAP/bronchitis = 14, PE = 5, and others = 12) were admitted to the ICU. Patients admitted to the ICU had lower pH (7.39 (IQR 7.28 to 7.49) vs. 7.43 (7.39 to 7.52), *P *< 0.01) and higher PaCO_2 _levels (5.5 kPa (IQR 4.5 to 11) vs. 5.2 kPa (IQR 4.4 to 7.8), *P *< 0.01) than patients not admitted. The prevalence of respiratory acidosis with hypoxemia was higher in patients admitted to ICU (20 of 91 patients, 41%) compared with patients not admitted (29 of 439 patients, 6.6%, *P *< 0.001). Multivariable logistic regression analysis identified pH as an independent ABGA parameter to predict ICU admission with an OR of 0.55 for every increase of pH of 0.1 (Table 2). From ROC analysis (area under curve 0.65, 95% CI 0.61 to 0.69), a pH less than 7.33 had a high specificity (90%) but low sensitivity (37%) to identify those patients in need for ICU care. The negative predictive value was 87%.

**Table 2:** Prognostic value of ABGA parameters to predict short-term and long-term outcome

ICU admission	OR	95% CI	AUC	95% CI	*P*-value
pH	0.552	0.43-0.71	0.647	0.61-0.69	< 0.001
PaCO_2_	1.309	1.15-1.50	0.591	0.55-0.63	0.001
PaO_2_	1.029	0.98-1.08	0.476	0.43-0.52	0.257
HCO_3_	1.00	0.96-1.05	0.504	0.46-0.55	0.956
Base excess	0.954	0.91-1.00	0.541	0.50-0.58	0.041
**In-hospital mortality**	**OR**	**95% CI**	**AUC**	**95% CI**	***P*-value**
pH	0.645	0.49-0.85	0.647	0.61-0.69	0.022
PaCO_2_	1.142	0.96-1.35	0.526	0.48-0.57	0.125
PaO_2_	1.050	1.00-1.11	0.576	0.53-0.62	0.133
HCO_3_	0.960	0.90-1.02	0.528	0.49-0.57	0.191
Base excess	0.943	0.89-1.00	0.546	0.50-0.59	0.045
**30 days mortality**	**OR**	**95% CI**	**AUC**	**95% CI**	***P*-value**
pH	0.602	0.46-0.79	0.650	0.61-0.69	< 0.001
PaCO_2_	1.205	1.04-1.40	0.562	0.52-0.61	0.015
PaO_2_	1.025	0.97-1.09	0.486	0.44-0.53	0.409
HCO_3_	0.977	0.92-1.03	0.504	0.46-0.55	0.416
Base excess	0.950	0.90-1.00	0.518	0.47-0.56	0.067
**12-month mortality**	**OR**	**95% CI**	**AUC**	**95% CI**	***P*-value**
pH	0.767	0.62-0.94	0.584	0.54-0.63	0.011
PaCO_2_	1.160	1.03-1.30	0.577	0.53-0.62	0.012
PaO_2_	1.040	1.00-1.09	0.518	0.47-0.56	0.071
HCO_3_	1.021	0.98-1.06	0.544	0.50-0.59	0.298
Base excess	1.022	0.98-1.06	0.559	0.52-0.60	0.296
**pH**	**OR**	**95% CI**	**AUC**	**95% CI**	***P*-value**
ICU admission	0.533	0.41-0.69	0.661	0.62-0.70	< 0.001
In-hospital mortality	0.621	0.44-0.88	0.662	0.61-0.71	0.007
30-day mortality	0.602	0.46-0.79	0.650	0.61-0.69	< 0.001
12-month mortality	0.767	0.62-0.94	0.584	0.54-0.63	0.011

Univariable logistic regression analysis of arterial blood gas analysis (ABGA) parameters to predict ICU admission, in-hospital mortality, 30-day mortality, and 12-month mortality. Multivariable logistic regression analysis of significant parameters from univariable analysis shows pH as independent predictor. Odds ratios (OR) with 95% confidence interval (95% CI) for ABGA parameters; OR are given for any increase of 1 kPa for partial pressure of arterial carbon dioxide (PaCO_2_), and partial pressure of arterial oxygen (PaO_2_), partial pressure of bicarbonate (HCO_3_), base excess and 0.1 for pH. Area under the curve (AUC) quantifying the diagnostic accuracy of the individual ABGA variables.

### Predictive value of pH at presentation for short-term outcome

The distribution of several baseline characteristics differed among the three groups stratified according to tertiles of pH (intertertile range 7.40 to 7.44; Table 3). Patients in the first tertile, with the lowest pH levels (pH ≤7.39), more often were current smokers, more often showed signs of decompensated heart failure, had higher respiration rates, had lower C-reactive protein levels, and presented with lower oxygen saturation at presentation.

**Table 3:** Baseline characteristics of patients stratified by tertiles of pH

Variable	pH ≤7.39 (*n *= 174)	pH 7.40- 7.44 (*n *= 177)	pH ≥7.45 (*n *= 179)	*P*-value
Age, years	74 (64-81)	74 (64-82)	75 (64-81)	0.99
Male sex, n (%)	62 (64)	80 (45)	75 (42)	0.18
Medical History, n (%)				
Coronary heart disease	76 (44)	67 (38)	65 (36)	0.33
Arterial hypertension	106 (61)	119 (67)	106 (59)	0.26
Stroke/peripheral arterial disease	37 (22)	34 (19)	30 (17)	0.53
Chronic obstructive pulmonary disease	80 (46)	85 (48)	72 (40)	0.31
Asthma	7 (4.0)	14 (7.9)	13 (7.3)	0.28
Any pulmonary disease	107 (62)	113 (64)	94 (53)	0.07
Pulmonary embolism	14 (8.0)	20 (11)	15 (8.4)	0.51
Deep venous thrombosis	16 (9.2)	23 (13)	13 (7.3)	0.18
Diabetes mellitus	45 (26)	40 (23)	35 (20)	0.37
Chronic kidney disease	51 (29)	43 (24)	33 (19)	0.06
Depressive disorder	22 (13)	27 (16)	24 (24)	0.69
Malignancy	28 (16)	30 (17)	38 (21)	0.40
Obesity	47 (29)	46 (27)	32 (18)	0.06
Smoking status, n (%)				0.01
never	14 (8.4)	21 (13)	33 (19)	
current	73 (44)	54 (32)	56 (31)	
former	54 (33)	72 (43)	71 (40)	
unknown	25 (15)	20 (12)	17 (9.1)	
Symptoms, n (%)				
Dyspnea				0.06
NYHA II	12 (6.9)	20 (11)	27 (15)	
NYHA III	79 (46)	80 (45)	89 (50)	
NYHA IV	83 (48)	77 (44)	63 (35)	
Thoracic pain	60 (35)	62 (35)	52 (29)	0.41
Orthopnea	68 (56)	104 (64)	103 (62)	0.61
Weight gain	20 (12)	25 (15)	18 (11)	0.50
Cough	91 (54)	116 (67)	106 (62)	0.04
Expectorant	68 (40)	78 (45)	81 (47)	0.40
Fever (> 38.5°C)	38 (22)	52 (30)	54 (32)	0.11
Medication, n (%)				
Diuretics	92 (53)	95 (54)	85 (48)	0.47
Nitrate	22 (13)	21 (12)	20 (11)	0.92
ACE inhibitors, angiotensin receptor blockers	69 (40)	88 (50)	74 (42)	0.14
Beta-blocker	48 (28)	49 (28)	56 (32)	0.67
Aspirin	52 (30)	62 (35)	56 (32)	0.57
Phenprocoumon/LMWH	39 (22)	42 (24)	38 (21)	0.87
Inhaled beta agonists	59 (34)	68 (38)	54 (30)	0.27
Inhaled steroids	46 (26)	47 (36)	54 (30)	0.65
Oral steroids	23 (13)	36 (20)	34 (19)	0.17
Clinical signs, n (%)				
Rales	81 (47)	83 (47)	87 (49)	0.92
Wheezing	61 (35)	65 (37)	48 (27)	0.10
Lower extremity edema	68 (39)	70 (40)	61 (34)	0.48
Jugular venous distension	47 (27)	24 (14)	32 (18)	0.01
Hepatojugular reflux	22 (13)	21 (12)	23 (13)	0.98
Vital status				
Systolic blood pressure (mmHg)	143 (127-162)	142 (126-165)	136 (122-158)	0.11
Diastolic blood pressure (mmHg)	88 (70-98)	86 (74-96)	82 (73-92)	0.23
Heart rate (beats per minute)	95 (78-110)	96 (82-111)	89 (76-110)	0.12
Respiration rate (breaths per minute)	25 (20-32)	24 (20-30)	22 (18-28)	0.03
Temperature (°C)	37.1 (36.5-37.8)	37.4 (36.7-39.7)	37.4 (36.8-38.0)	0.03
Oxygen saturation (%)	92 (87-96)	96 (92-98)	95 (93-98)	< 0.001
Body mass index (kg/m^2^)	26.2 (22.8-30.5)	26.3 (22.8-30.6)	25.3 (22.1-28.7)	0.20
Laboratory values				
Hemoglobin (g/L)	139 (122-155)	136 (123-150)	136 (125-146)	0.23
Leukocytes (10^9^/L)	10.6 (8.0-15.0)	10.0 (8.0-13.2)	10.5 (8.3-13.6)	0.40
C-reactive protein (mg/L)	15 (5-51)	21 (6-80)	27 (7-107)	0.02
Glomerular filtration rate (mL/min/1.73 m^2^)	57 (39-86)	64 (46-87)	65 (52-90)	0.06
B-type natriuretic peptide (pg/mL)	305 (93-810)	254 (52-712)	192 (65-760)	0.14
Discharge diagnosis, n (%)				
Acute heart failure	81 (47)	65 (37)	60 (34)	0.03
Exacerbation of COPD or asthma	37 (21)	43 (24)	38 (21)	0.73
Pneumonia or bronchitis	29 (17)	32 (18)	33 (18)	0.90
Pulmonary embolism	6 (3.4)	10 (5.6)	12 (6.7)	0.38
Anxiety disorder	1 (0.6)	4 (2.3)	13 (7.3)	< 0.001
Others	20 (12)	23 (13)	23 (13)	0.90

ACE, angiotensin-converting enzyme; COPD, chronic obstructive pulmonary disease; LMWH, low molecular weight heparin; NYHA, New York Heart Association. Data are presented as median and interquartile range, or number of patients (%).

Four hundred and sixty-two patients (87%) were admitted to the hospital. Table 4 shows that patients in the first tertile more often required admission to the ICU (28% vs 12% in the second, *P *< 0.001, and third tertile, *P *< 0.001), had a higher in-hospital mortality (14% vs 5% in the second, *P *= 0.002, and third tertile, *P *= 0.003), and a higher mortality after 30 days (17% vs 7% in the second, *P *= 0.002, and third tertile, *P *= 0.002).

**Table 4:** Outcome

Variable	pH ≤7.39 (*n *= 174)	pH 7.40-7.44 (*n *= 177)	pH ≥7.45 (*n *= 179)	*P*-value
Initial outcome				
Hospital admission, n (%)	158 (91)	151 (85)	153 (86)	0.12
ICU admission, n (%)	48 (28)	21 (12)	22 (12)	< 0.001
In-hospital mortality, n (%)	25 (14)	8 (4.5)	9 (5.0)	0.003
Short-term outcome				
30-day mortality, n (%)	30 (17)	12 (7.0)	12 (7.0)	0.002
Long-term outcome				
Mortality rate at 12 months (%)	37 (3.7)	28 (3.4)	23 (3.2)	0.005

Data are presented as number of patients (%), or cumulative mortality (%) (standard error).

### Predictive value of pH at presentation for long-term outcome

**Figure 2:**
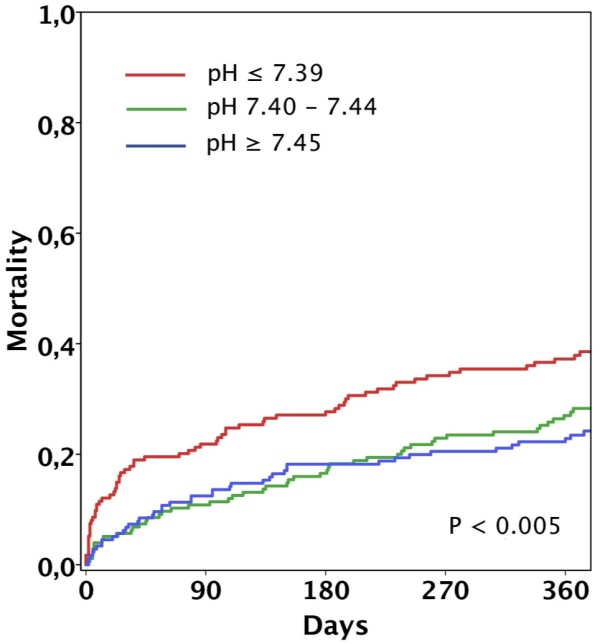
**12-month mortality of patients with acute dyspnea stratified by tertiles of pH**. 12-month mortality in all patients presenting with dyspnea (*n *= 530) according to tertiles of pH measured at presentation.

**Figure 3:**
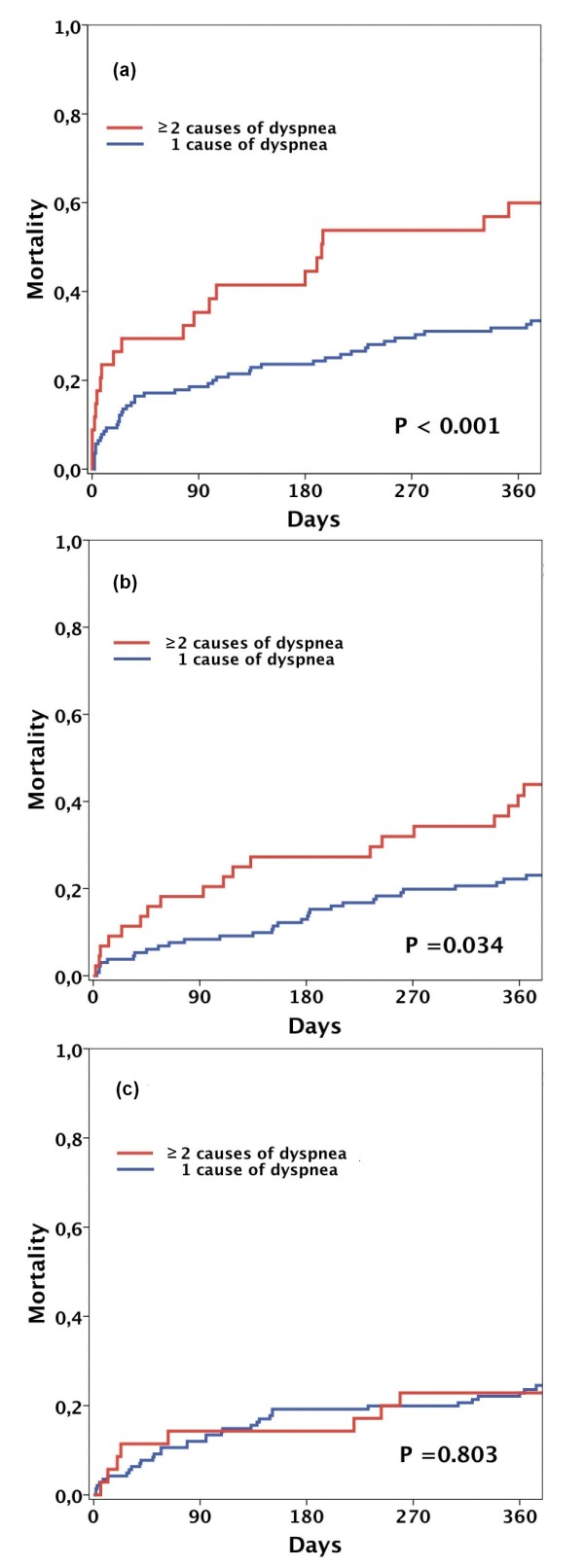
**12-month mortality of patients with multiple causes of dyspnea stratified by tertiles of pH**. 12-month mortality of patients presenting with multiple disorders responsible for acute dyspnea (*n *= 114) according to tertiles of pH measured at presentation (first tertile: pH ≤7.39, second tertile: 7.40-7.44, third tertile: ≥7.45).

During a median length of follow up of 425 (IQR 374 to 739) days in survivors, there were 175 deaths (33%) and a median time to death of 93 (20 to 211) days. After 12 months, 152 patients (29%) had died. Analysis based on pH at presentation showed a higher 12-month mortality rate for patients in the first tertile (37%) than in the second (28%) and third tertiles (23%, *P *= 0.005 by log-rank test; Figure 2). When analyzed according to the underlying disease, mortality in the first tertile remained higher both in patients with non-pulmonary causes of dyspnea (*P *= 0.040) and in patients with either exacerbated COPD/asthma or CAP/bronchitis (*P *= 0.005), respectively. Overall, mortality after 12-month follow-up was higher in patients with non-pulmonary causes of dyspnea (32% against 23%, *P *= 0.034). In patients who presented with more than one disorder responsible for dyspnea (41% against 25%, *P *= 0.008), 12-month mortality rates stratified by tertiles of pH were higher in the first tertile (*n *= 33, 60% (standard error (SE) 8.5), *P *< 0.001 by log-rank) and second tertile (*n *= 43, 44% (SE 7.6), *P *= 0.034) but not in the third tertile (*n *= 34, 23% (SE 7.1), *P *= 0.803; Figure 3).

Multivariable analysis showed that pH at presentation independently predicted 12-month mortality (HR 1.41, 95% CI, 1.11 to 1.79 for decrease of pH of 0.1, *P *= 0.005) in all studied patients presenting with acute dyspnea. Other independent predictors were previous use of oral diuretics, BMI and two or more causes of acute dyspnea. (Table 5). When analyses were repeated according to the underlying disorder, pH at presentation remained independent in the multivariable model in both patients with non-pulmonary (HR 1.41, 95% CI 1.02 to 1.92, *P *= 0.038) and pulmonary causes of dyspnea (HR 1.59, 95% CI 0.99 to 1.14, *P *= 0.043).

**Table 5:** Predictors of 12-month mortality in patients with acute dyspnea (significant variables only)

Variable	Hazard ratio	95% confidence interval	*P*-value
Previous use of oral diuretics	1.93	1.15-2.86	0.006
Body mass index (per increase of 1 unit)	0.93	0.90-0.97	< 0.001
≥2 causes of dyspnea	1.91	1.22-3.00	0.003
pH (per decrease of 0.1 unit)	1.41	1.11-1.79	0.005

Models were adjusted for age, sex, New York Heart Association (NYHA) class, history of coronary artery disease, history of chronic obstructive pulmonary disease, history of any pulmonary disease, history of chronic kidney disease, history of smoking, previous use of oral diuretics, previous use of inhaled beta agonists, systolic blood pressure, respiration rate, body mass index, two or more causes of dyspnea, and pH at admission.

### Subgroup analysis in patients with lactate values available

Lactate values were available in 298 patients (52%). In this subgroup, lactate was a significant predictor of diagnosis only in patients with AHF (OR 1.25, 95% CI 1.01 to 1.55; area under curve 0.573, 95% CI 0.51 to 0.63). As a prognostic value, lactate was not a significant predictor.

## Discussion

This prospective study in a large cohort of unselected patients presenting to the ED with acute dyspnea provides the following new information. First, ABGA parameters at presentation varied widely among patients with acute dyspnea. Second, used as a diagnostic test, ABGA parameters were useful neither to distinguish between patients with pulmonary disorders and other causes of acute dyspnea nor to identify specific disorders responsible for acute dyspnea. Acute diseases such as pulmonary or cardiac disorders seem to have a common final path leading to hypoxemia, increase of PaCO2, and increase of base excess irrespective of the original reason of the disorder. Only in patients with HV did pH and hypoxemia seem valuable. Third, pH measured at presentation was a significant predictor of short- and long-term outcome in dyspneic patients and was, independent from other predictors, associated with mortality, both in patients with pulmonary disorders and other causes of dyspnea.

These findings are of clinical importance as they question the usefulness of ABGA at presentation in patients presenting with acute dyspnea to the ED. As a diagnostic test, we found ABGA to be of very limited value in our cohort of dyspneic patients. Evaluating the prognostic value, pH was an independent predictor of mortality in both patients with pulmonary and other causes of dyspnea. Patients with a pH level of 7.39 or less showed a mortality rate after 12 months of 37% in our study and should receive particular attention. Arterial and/or venous blood gas analyses are valuable for risk stratification in the ED supporting disposition decisions or specific treatments such as non-invasive ventilation.

ABGA has been recommended for the clinical work up of dyspnea-related diseases [6-9]. In exacerbated COPD, elevated PaCO_2 _and decreased pH were found to be independent predictors of hospitalization and readmission [16,17]. In patients with PE, the diagnostic value of ABGA has been extensively investigated, as only around 35% with suspected PE actually do have the disease [10-12]. Results of these studies have been questioned for the inability of ABGA to sufficiently exclude PE, alone or in combination with other clinical data [13]. Accordingly, current diagnostic strategies in the evaluation of suspected PE have focused on probability scores, d-dimer testing and radiological imaging techniques rather than ABGA parameters [18]. In patients with unexplained exacerbation of COPD, it has been suggested that one out of four patients may in fact have PE [19,20]. A drop of PaCO_2 _of 0.7 kPa compared with baseline, together with a history of thromboembolism and the presence of malignancy have been found to predict PE in those patients [19]. This study supported earlier findings [21], but contrasted to results of the Prospective Investigation of Pulmonary Embolism Diagnosis (PIOPED) study, that could not identify differences between patients with and without COPD [22].

Dyspnea results from multiple interactions of signals in the limbic and autonomic nervous system, the motor cortex, and peripheral receptors [23]. It is a non-specific symptom and presents a clinical challenge even for experienced physicians. Most studies that evaluated the use of ABGA in patients with this unspecific symptom have focused on a single disorder, e.g. the diagnosis of suspected PE. Our study measured ABGA at presentation in 530 consecutive unselected patients presenting with acute dyspnea to the ED. We found ABGA to be of limited value in the management of this important group of patients in the ED. The diagnostic accuracy of ABGA provided useful to identify patients with HV but performed less well in other, more serious disease such as exacerbated COPD. Accordingly, our study does not favor measurement of ABGA as a diagnostic test in patients presenting with dyspnea. It is however important to recognize, that ABGA can be helpful in specific conditions as to control O_2 _saturation gap between transcutaneous oxygen saturation measurement and arterial oxygen saturation or in certain patients with hypoxemia.

Our study further showed that pH was the only ABGA parameter that, independently of other factors, predicted long-term outcome in patients presenting with acute dyspnea. Additionally, predictors of long-term prognosis were previous use of oral diuretics, BMI, and more than one cause of dyspnea. When analyzed according to the underlying disorder, pH remained an independent predictor of 12-month mortality in both patients with pulmonary and non-pulmonary causes of dyspnea. Patients with acute dyspnea from multiple causes had higher 12-month mortality than patients with a single. When analyzed according to tertiles of pH, long-term mortality was higher only in patients in the first and second tertile, independently increasing mortality in these patients. Long-term outcome is affected by both the severity of acute disease as well as comorbidity unrelated to the acute presenting condition. In our study, among them were factors related to the acute condition (≥2 causes of acute dyspnea and pH) as well as factors quantifying comorbidity (previous use of oral diuretics and BMI).

Previous studies have shown that pH is a predictor for the need of hospitalization [16] and in-hospital outcome [24,25] in exacerbated COPD and a predictor of in-hospital mortality in pneumonia and thus is an integral part of the clinical Pneumonia Severity Score (PSI) [26]. Recently, it was suggested that pH might also be an indicator of long-term survival [27,28]. Our results support these findings obtained in specific patient groups. Additionally, the present study expands the prognostic value of pH to the important group of patients presenting to the ED with acute dyspnea. In elderly patients (older than 65 years) presenting with acute respiratory failure to the ED, hypercapnia was among other variables a significant predictor of mortality [29]. In our cohort of unselected patients with a large majority of them being hospitalized (87%), pH was not only a predictor for long-term mortality but also predicted ICU admission, in-hospital mortality, and mortality after 30-day follow up. pH at presentation may therefore serve for the risk stratification of patients admitted for acute dyspnea.

Arterial puncture is not only painful and carries the risk vascular complications [30], it also may be difficult to perform, especially in an ED setting that might lack the calm that is needed for the procedure. Venous blood gas analysis has been proposed as a substitute for ABGA [31]. It has since been shown that venous pH has excellent correlation with arterial measurement of pH both in patients with exacerbated COPD [32,33] and other pulmonary and non-pulmonary disorders [32,34]. To a lesser extent, a clinically useful correlation has also been described for venous CO_2 _[34,35]. As expected, no correlation exists for PaO_2_. Taken together, the current evidence indicates that ABGA can be replaced by venous measurement of blood gases in the initial evaluation of patients in the ED.

Several limitations of the study should be considered. First, the decision to perform ABGA at presentation was exclusively at the discretion of the ED physician in charge based on the initial assessment of the patient. We did not use a standardized algorithm to decide if ABGA had to be performed. It is reasonable to believe that those patients who benefited most from ABGA were selected, for example a higher rate of ICU admission in patients with ABGA, and thus this bias should at least be conservative. Second, our prospectively defined endpoint was all-cause mortality. The classification of death in clinical practice is challenging and has been shown to be often inaccurate [36]. Third, we only examined patients with acute dyspnea presenting to the ED. Our results therefore do not negate the usefulness of ABGA in other settings, such as the titration of supplemental oxygen in patients with severe COPD and hypoxemia.

## Conclusions

Our study shows that ABGA at presentation is of limited value in the initial assessment of patients admitted to the ED for acute dyspnea. Used as a diagnostic parameter, ABGA proved not useful to distinguish between patients with pulmonary and non-pulmonary causes of acute dyspnea or to identify specific disorders responsible for acute dyspnea. Our study further showed that pH measured at presentation was a predictor of short- and long-term outcome in acutely dyspneic patients. Both in patients with pulmonary and other causes of acute dyspnea, the association of pH and mortality was independent of other predictors. pH might therefore be valuable for optimal risk stratification in this prevalent group of patients. However, the increasing evidence on the interchangeability of venous and arterial pH questions the need for ABGA measurement. Further prospective studies with consecutive venous blood gas analysis measurement at presentation are warranted to assess the prognostic value of pH in venous blood samples.

## Key messages

• ABGA parameters at presentation varied widely among patients with acute dyspnea.

• ABGA parameters were useful neither to distinguish between patients with pulmonary disorders and other causes of acute dyspnea nor to identify specific disorders responsible for acute dyspnea.

• pH measured at presentation was a significant predictor of short- and long-term outcome in acutely dyspneic patient.

• pH was, independently from other predictors, associated with mortality, both in patients with pulmonary disorders and other causes of acute dyspnea.

## Abbreviations

ABGA: arterial blood gas analysis; AHF: acute heart failure; BMI: body mass index; BNP: B-type natriuretic peptide; CAP: community-acquired pneumonia; CI: confidence interval; COPD: chronic obstructive pulmonary disease; ED: emergency department; HCO_3_: partial pressure of bicarbonate; HR: hazard ratio; HV: hyperventilation from anxiety disorder; IQR: interquartile range; NYHA: New York Heart Association; OR: odds ratio; PaCO_2_: partial pressure of carbon dioxide; PaO_2_: partial pressures of oxygen; PE: pulmonary embolism; ROC: receiver operating characteristics; SD: standard deviation; SE: standard error.

## Competing interests

The authors declare that they have no competing interests.

## Authors' contributions

EB and CM participated in study concept and design, acquisition of data, analysis and interpretation of data, drafting of the manuscript, and critical revision of the manuscript for important intellectual content. They also had full access to all of the data in the study and take responsibility for the integrity of the data and the accuracy of the data analysis. MP, BD, UA, CB, CH, MN, TB, NS, and TR participated in acquisition of data, analysis and interpretation of data and critical revision of the manuscript for important intellectual content. PS and AM participated in analysis, interpretation of data, drafting of the manuscript and critical revision of the manuscript for important intellectual content. All authors read and approved the final manuscript.

## Supplementary Material

Additional file 1 **Baseline characteristics**. Baseline characteristics of patients who received arterial blood gas analysis (ABGA) upon presentation with acute dyspnea to the emergency department and of patients who did not receive ABGA. Click here for file
