# Supplementary material for: Value of arterial blood gas analysis in patients with acute dyspnea: an observational study
Source: Crit Care. 2011 Jun 9;15(3):R145. doi: 10.1186/cc10268 (PMC3219017; doi:10.1186/cc10268)
Supplement: Additional file 1 — Baseline characteristics. Baseline characteristics of patients who received arterial blood gas analysis (ABGA) upon presentation with acute dyspnea to the emergency department and of patients who did not receive ABGA. [file cc10268-S1.DOC]

**ADDITIONAL FILE 1**

**Baseline Characteristics**

| **Variable** | **Patients with**  **ABGA (N=530)** | **Patients without ABGA (N=605)** | **P-value** |
| --- | --- | --- | --- |
| Age, years | 74 (64-81) | 76 (64-83) | 0.070 |
| Male sex, n (%) | 313 (59) | 307 (51) | 0.005 |
| Medical History, n (%) |  |  |  |
| *Coronary heart disease* | *208 (39)* | *235 (39)* | *0.890* |
| *Arterial hypertension* | *331 (63)* | *368 (61)* | *0.598* |
| *Stroke / Peripheral arterial disease* | *101 (19)* | *141 (24)* | *0.070* |
| *Chronic obstructive pulmonary disease* | *237 (45)* | *130 (22)* | *<0.001* |
| *Asthma* | *34 (6.4)* | *44 (7.3)* | *0.569* |
| *Any pulmonary disease* | *314 (59)* | *231 (38)* | *<0.001* |
| *Pulmonary embolism* | *49 (9.2)* | *40 (6.6)* | *0.100* |
| *Deep venous thrombosis* | *52 (9.8)* | *47 (7.8)* | *0.224* |
| *Diabetes mellitus* | *120 (23)* | *136 (23)* | *0.948* |
| *Renal failure* | *127 (24)* | *172 (28)* | *0.092* |
| *Depression* | *73 (14)* | *69 (12)* | *0.221* |
| *Malignancy* | *96 (18)* | *124 (21)* | *0.305* |
| *Obesity* | *125 (24)* | *110 (19)* | *0.024* |
| Smoking status, n (%)  *never*  *current*  *former*  *unknown* | *68 (13)*  *183 (36)*  *197 (39)*  *62 (12)* | *139 (24)*  *188 (33)*  *197 (34)*  *51 (8.9)* | <0.001 |
| Symptoms, n (%) |  |  |  |
| Dyspnea  *NYHA I*  *NYHA II*  *NYHA III*  *NYHA IV* | *0*  *59 (11%*  *248 (47)*  *223 (42)* | *10 (1.7)*  *86 (14)*  *293 (48)*  *216 (36)* | 0.003 |
| *Thoracic Pain* | *174 (33)* | *223 (37)* | *0.228* |
| *Orthopnea* | *191 (39)* | *223 (40)* | *0.789* |
| *Weight gain* | *63 (13)* | *113 (21)* | *0.001* |
| *Cough* | *313 (61)* | *294 (51)* | *0.001* |
| *Expectorant* | *227 (44)* | *203 (35)* | *0.003* |
| *Fever (>38.5°C)* | *144 (28)* | *91 (16)* | *<0.001* |
| Medication, n (%) |  |  |  |
| *Diuretics* | *272 (52)* | *340 (56)* | *0.101* |
| *Nitrate* | *63 (12)* | *96 (16)* | *0.053* |
| *ACE inhibitors, angiotensin receptor blockers* | *231 (44)* | *290 (48)* | *0.137* |
| *Beta-blocker* | *153 (29)* | *241 (40)* | *<0.001* |
| *Acetylsalicylic acid* | *170 (32)* | *206 (34)* | *0.470* |
| *Phenprocoumon / LMWH* | *119 (23)* | *163 (27)* | *0.078* |
| *Inhaled beta agonists* | *181 (34)* | *106 (18)* | *<0.001* |
| *Inhaled steroids* | *147 (28)* | *78 (13)* | *<0.001* |
| *Oral steroids* | *93 (18)* | *43 (7.1)* | *<0.001* |
| Clinical signs, n (%) |  |  |  |
| *Rales* | *251 (47)* | *298 (50)* | *0.455* |
| *Wheezing* | *174 (33)* | *113 (19)* | *<0.001* |
| *Lower extremity edema* | *199 (38)* | *244 (41)* | *0.295* |
| *Jugular venous distension* | *103 (20)* | *138 (23)* | *0.139* |
| *Positive hepatojugular reflux* | *66 (13)* | *108 (18)* | *0.010* |
| Vital status |  |  |  |
| *Systolic blood pressure (mm Hg)* | *140 (125-162)* | *140 (124-161)* | *0.391* |
| *Diastolic blood pressure (mm Hg)* | *85 (73-96)* | *84 (72-96)* | *0.851* |
| *Heart rate (beats per minute)* | *94 (79-109)* | *92 (76-109)* | *0.466* |
| *Respiration rate (breaths per minute)* | *24 (18-30)* | *20 (16-28)* | *0.001* |
| *Oxygen saturation (%)* | *95 (90-97)* | *97 (94-99)* | *<0.001* |
| *Temperature (°C)* | *37.3 (36.7-37.9)* | *37.2 (36.7-37.7)* | *0.119* |
| Laboratory values |  |  |  |
| *Hemoglobin (g/L)* | *13.7 (12.2-15.0)* | *13.2 (11.6-14.6)* | *0.001* |
| *Leukocytes (10^9^/L)* | *10.4 (8.2-14.4)* | *9.0 (7.0-11.7)* | *<0.001* |
| *Neutrophiles (10^9^/L)* | *7.9 (5.6-11.5)* | *6.5 (4.7-8.9)* | *<0.001* |
| *C-reactive protein (mg/L)* | *22 (6-79)* | *11 (3-41)* | *<0.001* |
| *Glomerular filtration rate (mL/min/ 1.73 m^2^)* | *63 (46-87)* | *61 (39-82)* | *0.058* |
| *B-type natriuretic peptide (pg/mL)* | *247 (70-762)* | *447 (101-1200)* | *<0.001* |
| Adjudicated diagnosis, n (%) |  |  |  |
| *Acute heart failure* | *206 (39)* | *351 (58)* | *<0.001* |
| *Exacerbation of COPD or asthma* | *118 (22)* | *77 (13)* | *<0.001* |
| *Pneumonia or bronchitis* | *94 (18)* | *52 (8.6)* | *<0.001* |
| *Pulmonary embolism* | *28 (5.3)* | *16 (2.6)* | *0.022* |
| *Anxiety disorder* | *18 (3.4)* | *19 (3.1)* | *0.809* |
| *Others* | *66 (13)* | *90 (15)* | *0.237* |
| Outcome, n (%) |  |  |  |
| *Hospital admission* | *462 (87)* | *489 (81)* | *0.004* |
| *ICU admission* | *91 (17)* | *50 (8.3)* | *<0.001* |
| *In-hospital mortality* | *42 (7.9)* | *36 (6.0)* | *0.153* |
| *30-day mortality* | *59 (11)* | *55 (9.0)* | *0.221* |
| *1-year mortality* | *175 (33)* | *182 (30)* | *0.123* |

ACE: Angiotensin-converting enzyme, NYHA: New York Heart Association, COPD: chronic obstructive pulmonary disease. Data are presented as median (interquartile range, IQR) or number of patients (%)**.**
